# Ralstonia pickettii Bacteremia: An Emerging Infection in a Tertiary Care Hospital Setting

**DOI:** 10.7759/cureus.5084

**Published:** 2019-07-05

**Authors:** Nosheen Nasir, Muneeba Ahsan Sayeed, Bushra Jamil

**Affiliations:** 1 Internal Medicine, Aga Khan University Hospital, Karachi, PAK

**Keywords:** emerging, bacteremia, ralstonia

## Abstract

Ralstonia species are Gram-negative bacilli that have increasingly been recognized as emerging nosocomial pathogens, particularly in immunocompromised hosts. Ralstonia pickettii is the most clinically important pathogen from the Ralstonia genus. Nosocomial outbreaks of Ralstonia pickettii infections brought about by the use of contaminated medical solutions, including saline, sterile water, as well as disinfectants, have been reported. There have been case reports of invasive infections with variable presentations.

Here, we describe three cases of Ralstonia pickettii bacteremia during a period of one year in a tertiary care hospital in Karachi, Pakistan. The first case was a 76-year-old male, known case of type 2 diabetes mellitus (DM), hypertension, and amyotrophic lateral sclerosis, who presented with complaints of burning micturition, hematuria, and fever. The patient had a history of multiple hospital admissions in the recent past. His blood culture was found to be positive for Ralstonia pickettii. A computed tomography scan of the kidneys, ureter, and bladder (CT KUB) was suggestive of pyelonephritis. The patient improved on intravenous meropenem. The second case was a 47-year-old man, who was admitted with a gunshot injury to the neck, resulting in complete cervical cord resection and mild hydrocephalus with intraventricular hemorrhage. The patient had a prolonged intensive care unit (ICU) stay, which was complicated by ventilator-associated pneumonia with Acinetobacter and central line-associated bloodstream infection (CLABSI) with Ralstonia pickettii. He was treated with meropenem and colistin but continued to deteriorate and expired. The third case was a 46-year-old lady, known case of end-stage renal disease (ESRD), who was admitted with prosthetic valve endocarditis. She had a prolonged hospital stay complicated by CLABSI with Ralstonia pickettii, improved on meropenem, but later died due to fungemia. Ralstonia pickettii is an emerging cause of nosocomial infection in patients, particularly those with a prolonged hospital stay, and can cause invasive and severe infections.

## Introduction

Ralstonia species are Gram-negative bacilli that have increasingly been recognized as emerging nosocomial pathogens, particularly in immunocompromised hosts [1]. Ralstonia pickettii, previously known as Burkholderia pickettii, is the most clinically important pathogen from the Ralstonia genus. Nosocomial outbreaks of Ralstonia pickettii infections brought about by the use of contaminated medical solutions, including saline, sterile water, as well as disinfectants, have been reported [2]. There have been case reports of meningitis, infective endocarditis, nosocomial pneumonia, and central line-associated bloodstream infection. Most of the infections occur in immunocompromised, particularly human immunodeficiency virus (HIV), patients and those with cystic fibrosis. The treatment of Ralstonia infection is still not well-defined in view of variable sensitivities, particularly to the carbapenems and aminoglycosides [1]. Here, we describe three cases of Ralstonia pickettii bacteremia, which are the first to be reported from Pakistan.

## Case presentation

Patient 1

A 76-year-old male, known case of type 2 diabetes mellitus (DM), hypertension, and amyotrophic lateral sclerosis, presented with complaints of burning micturition, hematuria, and fever. The patient was found to have increased total leukocyte count (TLC) and acute kidney injury. The patient had recently been admitted with infected bed sores and treated with piperacillin-tazobactam for Pseudomonas aeruginosa in pus from the bed sore. He was suspected with a nosocomial urinary tract infection and started empirically on meropenem. Urine culture grew Proteus mirabilis, which was pansensitive but the blood culture showed gram-negative rods, which were later identified to be Ralstonia pickettii that was sensitive to meropenem so that was continued. A computed tomography scan of the kidneys, ureter, and bladder (CT KUB) showed bilateral moderate hydronephrosis and hydroureter along with perinephric fat stranding. The urinary bladder appeared elongated in shape and thick-walled, along with minimal surrounding fat stranding. The overall findings were suggestive of reflux nephropathy secondary to bladder outlet obstruction. The patient improved on meropenem and was treated for 14 days.

Patient 2

A 47-year-old gentleman, with no prior comorbidities, was admitted with a gunshot injury to the neck, resulting in complete cervical cord resection and mild hydrocephalus with intraventricular hemorrhage. He was initially managed conservatively. He was noticed to be developing bilateral pleural effusion, which was heavily septated on the left. He required intubation and mechanical ventilation. He underwent video-assisted thoracoscopic surgery (VATS). The pleural fluid culture grew Acinetobacter spp and Pseudomona*s *aeruginosa​​​​​​. The patient had a prolonged intensive care unit (ICU) stay, which was complicated by ventilator-associated pneumonia with Acinetobacter and central-line associated bloodstream infection (CLABSI) with Ralstonia pickettii. He was treated with meropenem and colistin but had no meaningful neurological recovery and continued to deteriorate. It was decided by the family to withdraw support, and he expired.

Patient 3

A 46-year-old lady, known case of hypertension, end-stage renal disease requiring thrice weekly hemodialysis, and recent history of mitral valve replacement due to culture negative native valve infective endocarditis was admitted with non-ST elevation myocardial infarction. She required invasive ventilation and was admitted to the intensive care unit. She developed central line-associated bloodstream infection with Ralstonia pickettii. She was treated with intravenous meropenem. However, the patient continued to worsen clinically, as well as in terms of leukocytosis. Hence, intravenous levofloxacin was added to the treatment regimen. This led to an improvement in clinical parameters. However, the hospital course was prolonged and she eventually died of fungemia, again secondary to central line infection. Table 1 shows a summary of all three cases.

**Table 1:** SUMMARY OF CASES central line-associated bloodstream infection (CLABSI)

	Age/Gender	Source of Infection	Total Leukocyte Count (x10E9/L)		Treatment	Outcome
			Day 1	Day 3		
Patient 1	76/M	Pyelonephritis	20	13.8	Intravenous Meropenem & Colistin	Recovery
Patient 2	47/M	CLABSI	11.2	5.7	Intravenous Meropenem	Death
Patient 3	46/F	CLABSI	33	26	Intravenous Meropenem & Levofloxacin	Death

## Discussion

Ralstonia pickettii is an emerging Gram-negative opportunistic pathogen [1]. It is an aerobic non-fermenting bacillus, which is found in soil and water. Ralstonia pickettii has been rarely known to cause severe invasive infections, including pneumonia, endocarditis, osteomyelitis, meningitis, and septic arthritis [1-3]. It is a waterborne organism of low virulence, with a propensity to cause severe infections in the immunocompromised, resulting in significant mortality and morbidity [1]. Among our cases, two of the patients had chronic debilitating illnesses, which could have weakened their immune systems, predisposing them to infection with this organism. A number of cases have been attributed to contaminated solutions, e.g., saline solutions given as an intravenous infusion or utilized in wound cleaning or for endotracheal suctioning and reported from the contamination of dialysis water and extracorporeal membrane oxygenation therapy [4-7]. These have resulted in bloodstream, as well as respiratory, infections. However, in all of our cases, we could not identify any potential source of contamination. The underlying conditions reported to have been associated include alcoholic cirrhosis, diabetes mellitus, and intravenous drug abuse [1,8]. Two of our cases had hypertension, one had diabetes and end-stage renal disease while one patient had a prolonged ICU stay, which likely led to a nosocomial acquisition of organism. There have been reports of central line-associated infections with this pathogen and CLABSI was the identified source of infection in two of our cases also [9]. There have been several instances of outbreaks and because of the ubiquitous nature of the organism, differentiating between bacteremia and pseudo-bacteremia is important to avoid unnecessary treatment [4,10-11]. In vitro data suggest susceptibility to ceftriaxone but clinical failure has been reported and has been attributed to a lack of understanding of resistance mechanisms [12]. Other studies have shown in vitro susceptibility to quinolones, trimethoprim, and sulphamethoxazole [13]. Patients usually respond to intravenous third and fourth generation cephalosporins, which have been used as either monotherapy or in combination with amikacin or carbapenems [10]. We had used meropenem based on the susceptibility of our isolates. Colistin was used as part of the regimen in one of our cases because of multi-drug resistant (MDR) Acinetobacter pneumonia. One out of the three cases developed clinical failure with meropenem monotherapy, leading to the addition of levofloxacin based on culture and susceptibility, which resulted in improvement, as well as microbiological eradication evidenced by the clearance of blood culture. There have been reports of failure with meropenem where improvement was achieved with quinolones [14]. Treatment outcomes vary depending on the source of infection. Mortality in one of the series was reported to be around 7% [1]. Two out of three of our cases died, showing a poor outcome with infections caused by this organism. Our cases are the first to be reported from this region and highlight the importance of suspecting these infections in patients with healthcare-associated infections or who have had recent hospitalization or prolonged hospital stay requiring multiple interventions and invasive catheters. Table 2 summarizes the clinically significant bloodstream infections caused by Ralstonia pickettii.

**Table 2:** SUMMARY OF CASE REPORTS OF CLINICALLY SIGNIFICANT RALSTONIA PICKETTII BLOODSTREAM INFECTIONS central line-associated bloodstream infection (CLABSI)

Case report Authors	No of cases	Source of Infection	Treatment	Outcome
Tejera D. et al. [6]	2	Contaminated hemodialysis water	Meropenem, Piperacillin Tazobactam	Recovered
Kismet E. et al. [9]	2	CLABSI	Meropenem, Cefepime	Recovered
Orme J. et al. [2]	1	Infective endocarditis	Trimethoprim-sulfamethoxazole and levofloxacin	Died
Strateva T. et al. [15]	1	Contaminated hemodialysis water	Levofloxacin	Recovered
Khajuria A. et al. [16]	1	Surgical site infection	Tigecycline	Recovered

## Conclusions

Ralstonia pickettii should be considered an important pathogen causing hospital-acquired infections, particularly among patients who are immunocompromised, as it can have a poor prognosis.
